# CCDC114, DNAI2 and TOP2A involves in the effects of tibolone treatment on postmenopausal endometrium

**DOI:** 10.1186/s12905-020-01156-6

**Published:** 2021-06-11

**Authors:** Yanhua Lv, Yanqing Liu, Yueqiang Wang, Fanrong Kong, Qiuxiang Pang, Guirong Hu

**Affiliations:** 1 Department of Gynecology, Affiliated Hospital of Jining Medical University, Jining, 272000 Shandong China; 2 Department of General Medicine, Jining No. 1 People’s Hospital, Jining, 272011 Shandong China; 3 Department of Internal Medicine-Cardiovascular, Affiliated Hospital of Taishan Medical University, Taian, 271000 Shandong China; 4 Laboratory of Developmental and Evolutionary Biology, School of Life Sciences, Shandong University of Technology, Zibo, 255049 Shandong China; 5 Department of Obstetrics and Gynecology, People’s Hospital of Jiaxiang County, No. 188 Yingfeng Road, Jiaxiang, Jining, 272400 Shandong China

**Keywords:** Differentially expressed genes, Function and pathway analysis, Gene–drug investigation, Postmenopausal women, Protein–protein interaction network

## Abstract

**Background:**

This study aimed to explore the molecular mechanisms of tibolone treatment in postmenopausal women.

**Methods:**

The gene set enrichment profile, GSE12446, which includes 9 human endometrial samples from postmenopausal women treated with tibolone (tibolone group) and 9 control samples (control group), was downloaded from GEO database for analysis. Differentially expressed genes (DEGs) in tibolone vs. control groups were identified and then used for function and pathway enrichment analysis. Protein–protein interaction (PPI) network and module analyses were also performed. Finally, drug–target interaction was predicted for genes in modules, and then were validated in Pubmed.

**Results:**

A total of 238 up-regulated DEGs and 72 down-regulated DEGs were identified. These DEGs were mainly enriched in various biological processed and pathways, such as cilium movement (e.g., CCDC114 and DNAI2), calcium ion homeostasis, regulation of hormone levels and complement/coagulation cascades. PPI network contained 368 interactions and 166 genes, of which IGF1, DNALI1, CCDC114, TOP2A, DNAH5 and DNAI2 were the hue genes. A total of 96 drug–gene interactions were obtained, including 94 drugs and eight genes. TOP2A and HTR2B were found to be targets of 28 drugs and 38 drugs, respectively. Among the 94 obtained drugs, only 12 drugs were reported in studies, of which 7 drugs (e.g., epirubicin) were found to target TOP2A.

**Conclusions:**

CCDC114 and DNAI2 might play important roles in tibolone-treated postmenopausal women via cilium movement function. TOP2A might be a crucial target of tibolone in endometrium of postmenopausal women.

## Background

The emergence of various characteristics of metabolic syndrome are implicated in the process of premenopause transition to postmenopause [1]. As women age and estrogen levels decrease, coronary artery disease, osteoporosis, and endometrial diseases are major causes of mortality and morbidity [2, 3]. Observational studies have suggested that postmenopausal hormone treatments, such as estrogen replacement therapy, are effective in preventing endometrial disease [4]. However, a previous study found that while estrogen treatment reduced the occurrence of heart disease and fractures in postmenopausal women, long-term treatment with estrogen promoted the risk of endometrial cancer [5]. Unopposed estrogen treatment raises the risk of endometrial hyperplasia and the succeeding carcinoma [6].

Tibolone is a synthetic steroid used for hormone replacement therapy in postmenopausal women [7]. Tibolone shows tissue-selective properties that have estrogenic activity in the vagina, brain, and bone, but not in endometrial and breast tissues [8]. Tibolone does not stimulate the endometrium and does not induce endometrial hyperplasia in postmenopausal women. In comparison with estrogen–progestogen therapy, tibolone is associated with less vaginal bleeding [9, 10]. It had been reported that low doses of tibolone had favorable effects on postmenopausal endometrium by inducing progestogenic properties to create a balance between pro- and anti-apoptotic actions [11]. Nevertheless, there is a requirement to further investigate the molecular mechanisms of action of tibolone in postmenopausal endometrium.

Hanifi-Moghaddam et al., generated the GSE12446 microarray dataset, which compares gene expression between endometrial samples from postmenopausal women after treatment with tibolone for 21 days and endometrium from women treated with estradiol-only + medroxyprogesterone acetate [12]. They observed a close relationship between tibolone treatment and gene expression in endometrium from both groups of women. However, the study did not examine the detailed molecular mechanisms of tibolone action in postmenopausal women.

Herein, we used gene expression data in GSE12446 (of the 36 samples, only data from 9 tibolone-treatment samples and 9 control group samples were used) to perform bioinformatics analysis to further investigate the molecular mechanisms of tibolone action in postmenopausal endometrium [12]. Differentially expressed gene (DEGs) were identified and used in function and pathway enrichment analysis and protein–protein interaction (PPI) network analysis. Finally, drug–gene interactions prediction was performed to identify drugs with potential for use in postmenopausal women. The current study will provide a theoretical basis for further research regarding tibolone-induced gene expression changes in the endometrium of postmenopausal women, and provide potential targets for use in clinical treatment (Additional file 1: Figure S1 shows the workflow of this study).

## Methods

### Microarray data

The microarray dataset “GSE12446” was downloaded from the Gene Expression Omnibus (GEO, http://www.ncbi.nlm.nih.gov/geo/) database. The samples were pure endometrial tissue collected from patients who underwent vaginal hysterectomy for treatment of prolapse. Of the 36 samples in total, the gene expression data of 9 samples from the tibolone group (2.5 mg oral tibolone administered daily, starting 21 days prior to surgery) and 9 samples from the control group (no hormonal treatment) were used. The microarray platform used was the GPL570 (HG-U133_Plus_2) Affymetrix Human Genome U133 Plus 2.0 Array.

### Data processing and DEGs screening

CEL raw data was preprocessed based on a robust multi-array average (RMA) method of affy (version: 1.50.0) package in R software [13]. Probe IDs were converted to gene symbols based on the annotation files from the platform. The probes that did not match with a gene symbol were removed, and the mean value was selected as the final expression value when multiple probes matched to only one gene symbol. Differential gene expression analysis between tibolone-treated and control group samples was performed using the Bayesian method in Linear Models For Microarray Data (limma) [14]. The Benjamini and Hochberg method was used to perform multiple testing correction. Adjusted *P* value (adj.*P*.Value) < 0.05 and log-fold change (FC) ≥ 1 were selected as the threshold values for DEGs screening. Screened DEGs were then visualized using heat maps and volcano plots.

### Function and pathway enrichment analysis

DAVID software (version: 6.8, https://david-d.ncifcrf.gov/) [15] was used to examine enriched functional pathways in the screened DEG lists based on gene ontology (GO) annotations [16] and Kyoto Encyclopedia of Genes and Genomes (KEGG) pathways [17]. Significantly enriched GO terms and KEGG pathways were defined using a threshold of *P* < 0.05 and enriched gene count ≥ 3.

### PPI network construction and module analysis

To explore interactions between proteins encoded by DEGs, DEGs were uploaded to the STRING database (version: 10.0) [18] to extract PPIs using parameters set to: median confidence (score) = 0.4, species = homo. The resulting PPI networks were visualized using Cytoscape (version: 3.4.0). CytoNCA software [19] was used to analyze the topology properties of nodes in the networks. MCODE (Version1.5.1) [20], a plug-in of Cytoscape software, was used to conduct cluster analysis and identify functional modules from the PPI network using default parameters. Functional modules with a modules score ≥ 5 were considered significant. ClusterProfiler (version: 3.8.1) software was used to further investigate genes in significant functional modules for enriched biological processes using GO annotations and KEGG pathways. The Benjamini and Hochberg method was used to perform multiple testing correction. The significantly enriched terms were selected with the cut-off of adj.*P*.value < 0.05 and count ≥ 2.

### Prediction of drug–gene interactions

Based on the online GDIdb [21]database (version: 3.0), the gene–drug interactions were predicted based on the genes in significant modules using the following parameter settings: Preset Filters, FDA Approved; Advanced Filters, Source Databases, 20 of 20; Gene Categories, 41 of 41; Interaction Types, 51 of 51. Drug–gene interactions were visualized using Cytoscape software. These drug–gene interactions were further validated by searching Pubmed using the key words of ‘Postmenopausal’, and ‘Endometrium,’ and “drug names” using Biopython in python software.

## Results

### DEGs between tibolone group and control group

**Fig. 1:**
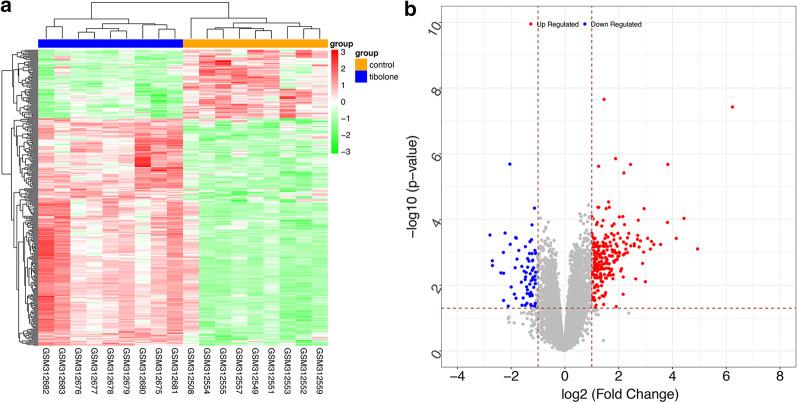
Heat map and volcano plot of differentially expressed genes in tibolone-treated samples compared to untreated control samples of endometrium from postmenopausal women. **a** Heat map of DEGs. Yellow bar represents control samples, blue bar represents tibolone treatment samples, green represents down-regulated genes, red represents up-regulated genes. Darker color shades represent greater statistical significance. **b** Volcano plot of DEGs. Blue nodes represent down-regulated genes, red nodes represent up-regulated genes, grey nodes represent non-differentially expressed genes. The x*-*axis represents log fold-change values, the y-axis represents –log *P* values

A total of 238 significantly upregulated DEGs and 72 significantly down-regulated DEGs were identified in the tibolone group compared to the control group (*P* < 0.05, ≥ twofold change, Fig. 1a) and used for subsequent analysis. As shown in heatmap of DEGs, the samples in different groups could be obviously distinguished by up-regulated and down-regulated genes. A volcano plot of the screened DEGs is shown in Fig. 1b.

### Enrichment analysis of DEGs

**Fig. 2:**
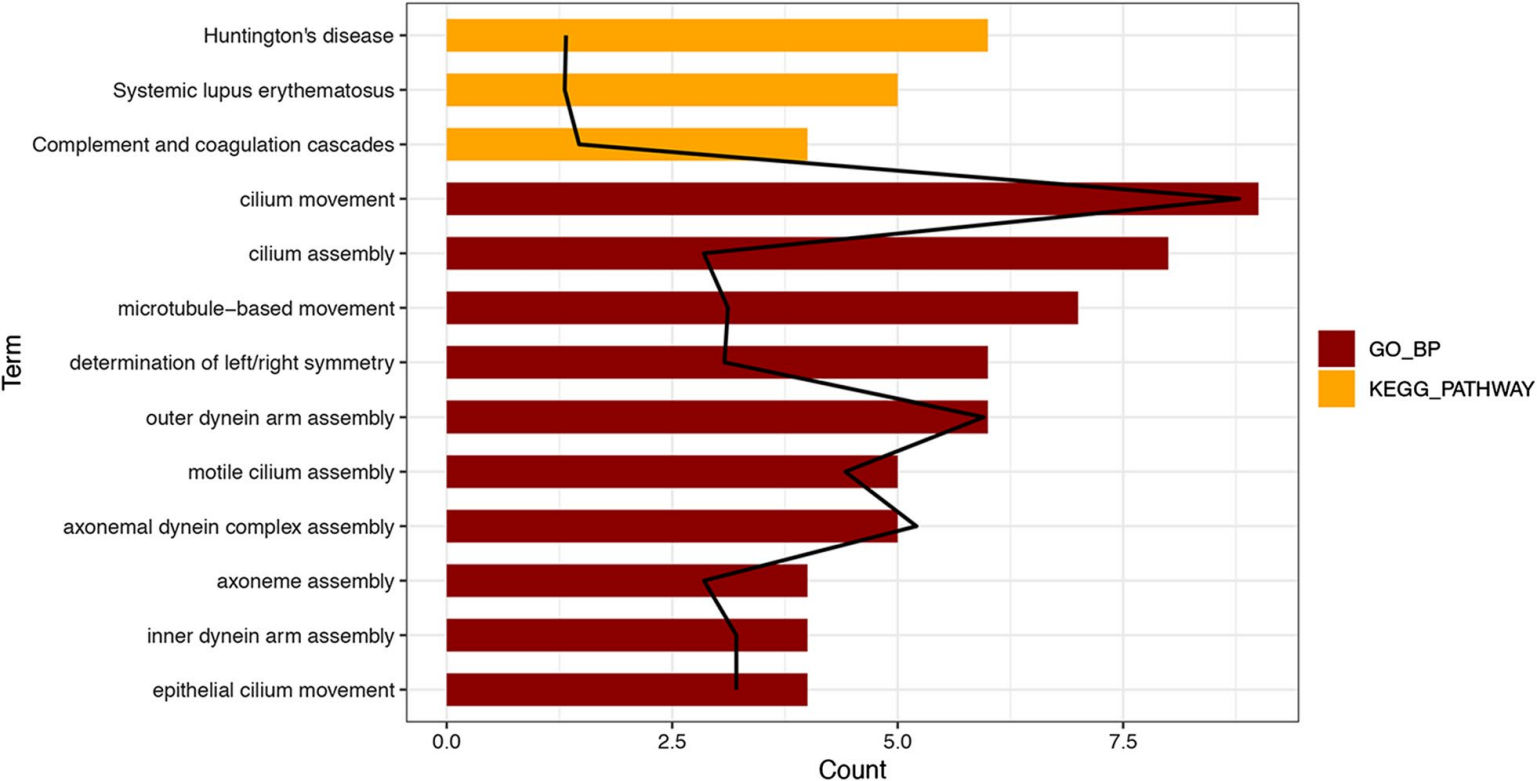
Function and pathway enrichment analysis of the differentially expressed genes. Enrichment analysis indicating the biological function and pathways likely involved based on DEGs in response to tibolone in postmenopausal endometrium. Dark red represents the top 10 functions; yellow represents the top 3 pathways. Black lines represent −log *P* values. The x-axis represents the number of genes in a certain block; the y-axis lists the function or pathway

The DEGs were significantly enriched in 36 GO-biological processes and 3 KEGG pathways in the functional enrichment analysis (Additional file 2: Table S1). The three KEGG pathways and the top 10 GO-biological processes were shown in Fig. 2. These DEGs were predominantly involved in functions like cilium movement (GO: 0003341, Genes: Coiled-Coil Domain Containing (CCDC) 114, Dynein Axonemal Intermediate Chain (DNAI) 2, etc.), cilium assembly (GO: 0042384, Gene: CCDC113, DNAI2, etc.) and microtubule-based movement (GO: 0007018, Gene: Dynein Axonemal Heavy Chain (DNAH) 9, Kinesin Family Member (KIF) 4A, etc.). Pathway analysis showed that these DEGs were enriched in pathways including Huntington’s disease (hsa04610), systemic lupus erythematosus (hsa05016) and complement/coagulation cascades (hsa05322).

### PPI network and module investigation

**Fig. 3:**
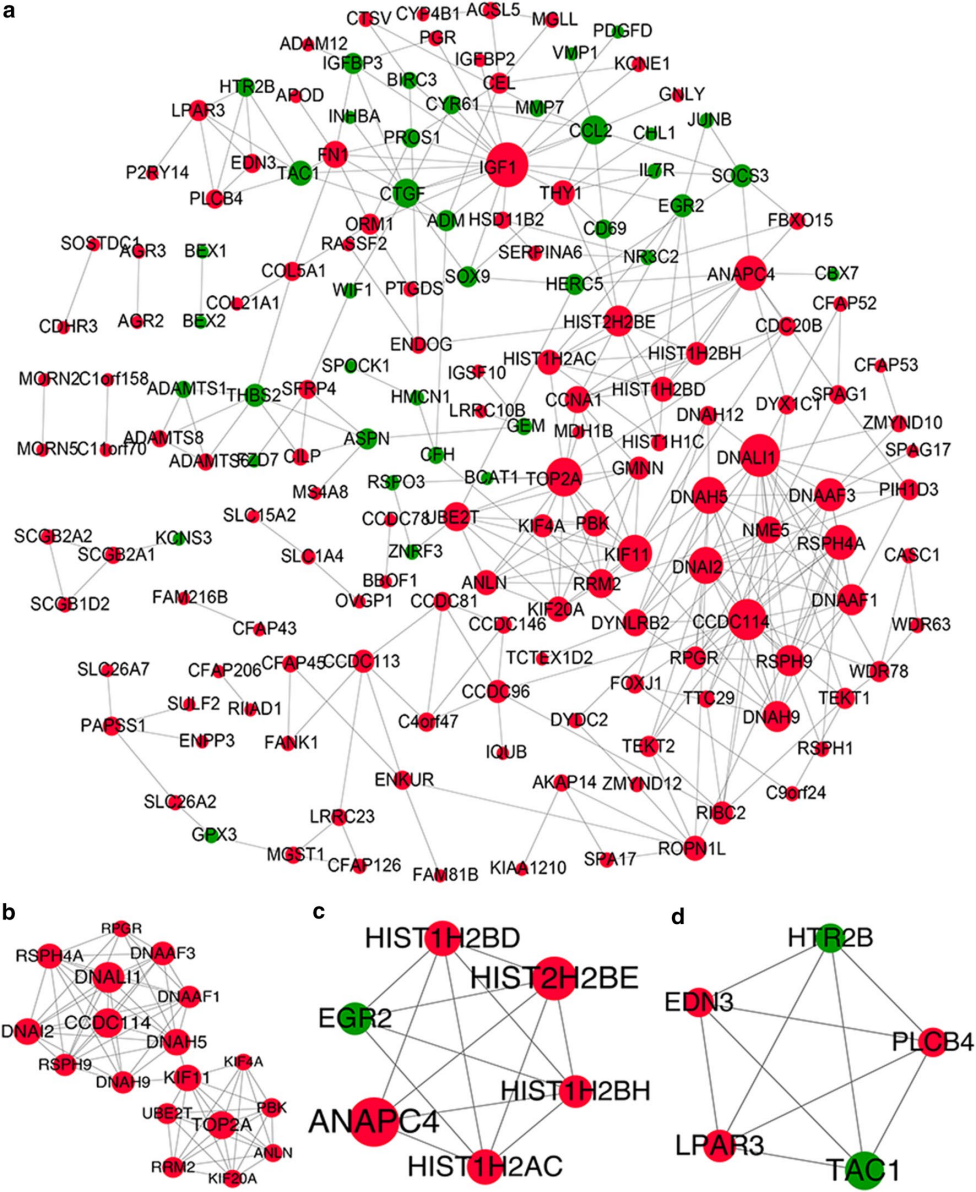
Protein–protein interaction network and modules. PPI network analysis shows the known and predicted interactions (including physical and functional associations) between proteins encoded by DEGs. Theses connectivity networks are helpful in fully understanding biological phenomena **a** PPI network. **b** Module 1. **c** Module 2. **d** Module 3. Red circles represent up-regulated genes; green circles represent down-regulated genes; the larger the node size, the higher the degree value. Lines between two nodes represent an interaction

To further derive effective information from the identified DEGs, PPI networks were constructed based on the relationships among proteins that the DEGs are known to encode. A total of 368 protein interactions and 166 nodes were revealed (Fig. 3a). In the network topological properties analysis, the degree of each node were analyzed (Additional file 3: Table S2). It could be seen that insulin like growth factor 1 (IGF1, degree = 17), DNALI1 (degree = 16), CCDC114 (degree = 15), DNA topoisomerase II alpha (TOP2A, degree = 14), DNAH5 (degree = 13), DNAI2 (degree = 13) and Kinesin Family Member 11 (KIF11, degree = 13) were hub nodes with higher degree in PPI network.

**Fig. 4:**
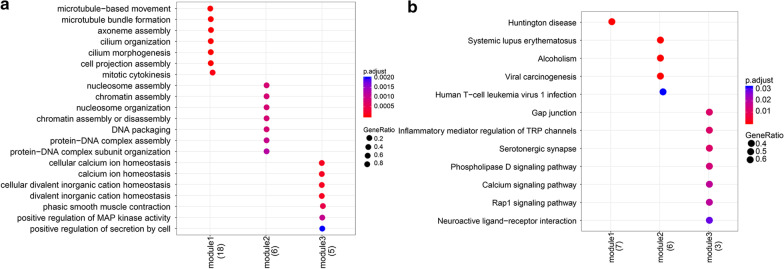
Function and pathway enrichment analysis of differentially expressed genes in modules. Significant modules identified from PPI network by cluster analysis indicate the protein complexes or functional modules with biological significance. Enrichment analysis of the genes in these modules reveals their exact biological functions. **a** The top 7 functions of DEGs in each module; the x-axis represents the different modules, the y-axis represents function. **b** Pathways enriched by DEGs in each module. The x-axis represents the different modules; the y-axis represents enriched pathways

Furthermore, three significant modules with score ≥ 3 were identified from PPI network(Fig. 3b–d). Module 1 contained 18 up-regulated genes, including TOP2A, KIF11, CCDC114 and DNAI2, etc. The genes in Module 1 were significantly enriched in 14 GO terms and only one KEGG pathway, such as microtubule-based movement (GO: 0007018), mitotic cytokinesis (GO: 0000281) and Huntington’s disease (hsa04610). Module 2 contained 6 DEGs (ANAPC4, HIST2H2BD, HIST2H2BE, etc.), and these genes were mainly enriched in 7 GO terms and 4 KEGG pathways, such as nucleosome assembly (GO: 0006334), protein-DNA complex assembly (GO: 0065004), and pathways like systemic lupus erythematosus pathology (hsa05016). Module 3 contained 5 DEGs, and these DEGs were predominantly enriched in 24 GO terms and 7 KEGG pathways, such as calcium ion homeostasis (GO: 0055074), regulation of hormone levels (GO: 0010817), gap junction pathways (hsa04540), and calcium signaling pathway (hsa04020) (Fig. 4, Additional file 4: Table S3).

### Drug–gene interaction investigation

**Fig. 5:**
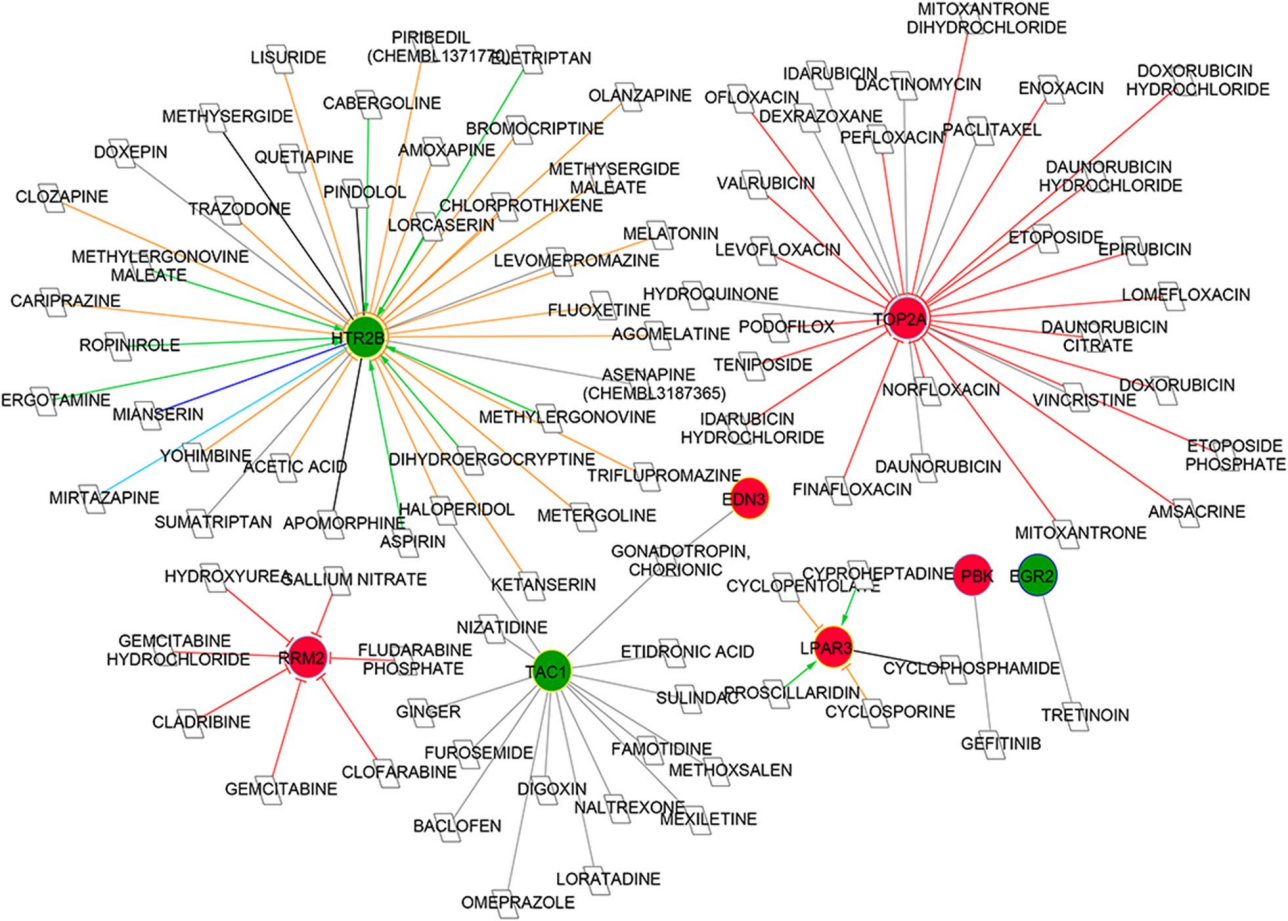
Drug–gene interaction network based on differentially expressed genes and related drugs. The gene–drug interactions retrieved from the DGIdb database shows genes that are targeted therapeutically or prioritized for drug development. White parallelograms represents predicted drugs, red circles represents up-regulated genes, green circles represent down-regulated genes. The outer circle color indicates the gene belongs to the module: module 1, light purple; module 2, dark blue; module 3, bright yellow. Grey connections represent unknown interaction relationships. Red T connections represent inhibitor relationships, yellow T connections represent antagonist relationships, green arrows represent agonist and partial agonist relationships; black connections represent agonist/antagonist relationships; light blue connections represent binder relationships; dark blue connections represent binder/antagonist relationships

Drug–gene interactions were predicted for the 29 genes in significant modules. A total of 96 drug–gene interactions were obtained, including 94 drugs and eight genes (Fig. 5, Additional file 5: Table S4). Among the eight genes, there were three down-regulated genes and five up-regulated genes, including ribonucleotide reductase regulatory subunit M2 (RRM2), TOP2A, PDZ binding kinase (PBK), and 5-hydroxytryptamine receptor 2B (HTR2B). TOP2A was found to target by 28 drugs and HTR2B was a target of 38 drugs. These interactions included 28 inhibitor relationships, 21 antagonist relationships, 10 agonist relationships, 4 agonist or antagonist relationships, 1 binder relationship, 1 binder/antagonist relationship, 1 partial agonist relationship and 30 unknown relationships. Such as, epirubicin was predicted to be an inhibitor for TOP2A, and eletriptan was predicted to be an agonist for HTR2B.

### Validation of drugs

**Fig. 6:**
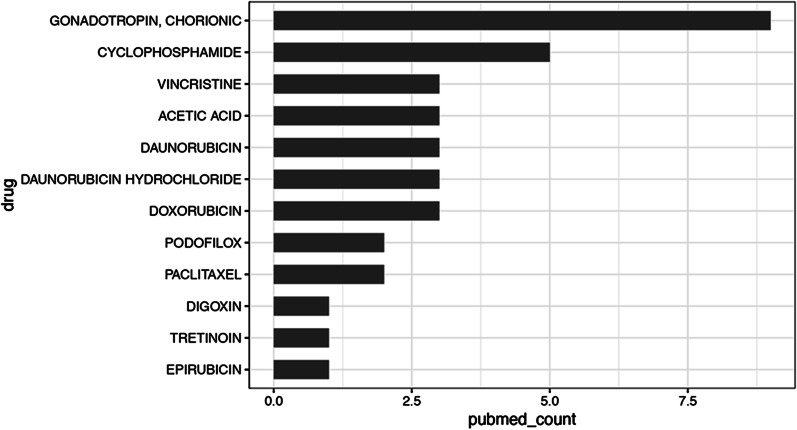
Novel drug predications based on literature review. Results of a key word search of the PubMed database. Key words included ‘postmenopausal’ and ‘endometrium,’ as well as drug names. Drugs reported in 0 to 5 studies relevant to post-menopause and endometrium were considered novel drugs for investigation. The x-axis represents the number of published documents; the y-axis indicates drug names

The obtained drug–gene interactions above were further validated by searching Pubmed using the key words of ‘Postmenopausal’, and ‘Endometrium,’ and “drug names” using Biopython in python software. Among the 94 drugs, only 12 drugs were reported in studies (Fig. 6, Additional file 6: Table S5), including epirubicin, Tretinoin, Digoxin, Paclitaxel, Podofilox, Doxorubicin, Daunorubicin hydrochloride, Daunorubicin, Acetic acid, Vincristine, Cyclophosphamide and Gonadotropin chorionic.

## Discussion

Although tibolone is commonly used as an alternative for estrogen replacement therapy, the detailed molecular mechanism of action of tibolone and its clinical application in postmenopausal women are still unclear. In the present bioinformatics study, a total of 238 up-regulated DEGs and 72 down-regulated DEGs were identified and investigated. These DEGs were predominantly assembled in functions such as cilium movement, calcium ion homeostasis, regulation of hormone levels, complement/coagulation cascades, etc. IGF1, DNALI1, CCDC114, TOP2A, DNAH5 and DNAI2 were the hue genes in PPI network. Furthermore, TOP2A and HTR2B were found to be targets of 28 drugs and 38 drugs, respectively. Among the 94 obtained drugs, only 12 drugs were reported in studies, of which 7 drugs (for example, epirubicin) were found to target TOP2A. These suggested that TOP2A might be a crucial targets in endometrium of drug treated in postmenopausal women.

Cilium movement is closed related to the biological functions of the human oviduct [22]. The frequency of cilium movement appears to increase in the late follicular phase compared to that in both the early follicular phase and the luteinizing phase, and can be reduced by progesterone receptor regulation [23]. Human endometrium consists of stromal and epithelial cells, of which, epithelial cells include secretory and ciliated cells. Ciliogenesis in endometrium is driven by estrogen signaling [24]. Changes in the expression of cilia-related genes has been reported to be associated with differentiation, nodal metastasis and recurrence of endometrial cancer [25].

Cilium-associated CCDC114 participates in cilium movement function in vivo [26]. Mutations in CCDC114 are a cause of primary ciliary dyskinesia (PCD) [27]. A mutation in the CCDC114 gene causes PCD with normal fertility in humans [28]. Although CCDC114 has been shown to be essential for motile cilia in many diseases, its effects in postmenopausal women has not been well studied. DNAI2 mutation is another important factor influencing the function of cilium movement [29]. DNAI2 is down-regulated in the oviduct of non-obese diabetic mice when compared with that in healthy mice [30]. In mouse ovaries, the expression of DNAI2 was detected at high levels in vivo on day 10, with a subsequent decrease on days 15 and 20 [31]. In the current study, GO functional analysis of DEGs identified cilium movement function as an enriched pathway that both CCDC114 and DNAI2 were up-regulated in. Thus, we speculated that CCDC114 and DNAI2 may have a role in cilium movement function in postmenopausal women treated with tibolone.

Epirubicin (4′-epi-doxorubicin) is an anthracycline antibiotic, a doxorubicin analog differing by epimerization of the hydroxyl group at position 4′ of the aminosugar moiety. Similar to doxorubicin, epirubicin shows anti-tumor activity by binding to DNA and inhibiting DNA synthesis and function [32, 33]. Epirubicin has been used as a first-line medicine for cancer in postmenopausal women [34]. Although there is a dose–response effect for epirubicin in clinical treatment for postmenopausal patients [35], the combination of epirubicin and other drugs, including docetaxel and tamoxifen, can enhance the effects of epirubicin [36, 37]. However, whether epirubicin is suitable for the treatment of postmenopausal women is still unclear due to multiple drug resistance [38]. Interestingly, the expression of the TOP2A gene is closely related to epirubicin and drug-resistance [39]. TOP2A alterations are a predictive marker for epirubicin sensitivity in clinical treatment [40]. TOP2A protein levels are used as a predictor of response to epirubicin as a neoadjuvant treatment for breast cancer [41]. TOP2A was also reported to be a predictive biomarker in endometrial cancer patients receiving taxane-containing adjuvant chemotherapy [42]. In the current study, drug–gene interaction analysis showed that there was a negative association between up-regulated TOP2A and epirubicin. Thus, we speculate that TOP2A might be a crucial target to investigate the potential effect of epirubicin in postmenopausal women. In addition, among the 94 obtained drugs, only 12 drugs were reported to play role in endometrium of in postmenopausal women in studies, of which 7 drugs (for example, epirubicin) were found to target TOP2A. These suggested that TOP2A was a crucial target. Moreover, TOP2A was a significant DEGs in tibolone treated endometrium of postmenopausal women. From all above, we speculate that epirubicin may have potential clinical value in the treatment for endometrium of postmenopausal women. However, the role of epirubicin and its clinical value for endometrium of postmenopausal women should be confirmed by more in vitro and in vivo experiments.

Despite these exciting new findings, there remains some limitations in this study. (1) we preliminarily investigated the DEGs in tibolone treated endometrium of postmenopausal women and their involved biological processes and pathways. The expression of these DEGs should be confirmed in clinical samples by PCR and Western blot, and their potential functions should be investigated by a series of functional experiments. (2) Only nine samples in each group were included. Further investigation based on a large sample size was required to investigate the molecular mechanism. (3) Several drugs were predicted to target TOP2A, which was a significant DEGs in tibolone treated endometrium of postmenopausal women. It was needed to further investigate whether TOP2A was a direct target of tibolone in endometrium of postmenopausal women and whether these drugs could replace tibolone to target TOP2A in postmenopausal women.

## Conclusion

In conclusion, expression changes of multiple genes were found in endometrium of tibolone treated postmenopausal women. These genes were implicated in cilium movement, calcium ion homeostasis, regulation of hormone levels and complement/coagulation cascades. CCDC114 and DNAI2, which both participate in cilium movement function, are upregulated in postmenopausal women treated with tibolone. TOP2A might be a crucial target of tibolone in endometrium of postmenopausal women.

## Supplementary information

**Additional file 1: Figure S1.** The workflow of this study.

**Additional file 2: Table S1.** The enriched biological processes terms and KEGG pathways for DEGs.

**Additional file 3: Table S2.** Results of topological properties analysis for nodes in PPI network.

**Additional file 4: Table S3.** The enriched GO terms and KEGG pathways for genes in significant modules identified from PPI network.

**Additional file 5: Table S4.** The obtained drug-gene interactions information from GDIdb database.

**Additional file 6: Table S5.** Validation of the predictive drugs in Pubmed.

## Data Availability

The datasets used and/or analyzed during the current study are available from the corresponding author on reasonable request.

## Abbreviations

DEGs: Differentially expressed genes
PPI: Protein-protein interaction network
SLE: Systemic lupus erythematosus
CCDC: Coiled-Coil Domain Containing
DNAI: Dynein Axonemal Intermediate Chain
BP: Biological process
RRM2: Reductase regulatory subunit M2
TOP2A: DNA topoisomerase II A
PBK: PDZ binding kinase
