# Supplementary figures and images for: CCDC114, DNAI2 and TOP2A involves in the effects of tibolone treatment on postmenopausal endometrium
Source: BMC Womens Health. 2021 Jun 11;21:240. doi: 10.1186/s12905-020-01156-6 (PMC8194000; doi:10.1186/s12905-020-01156-6)

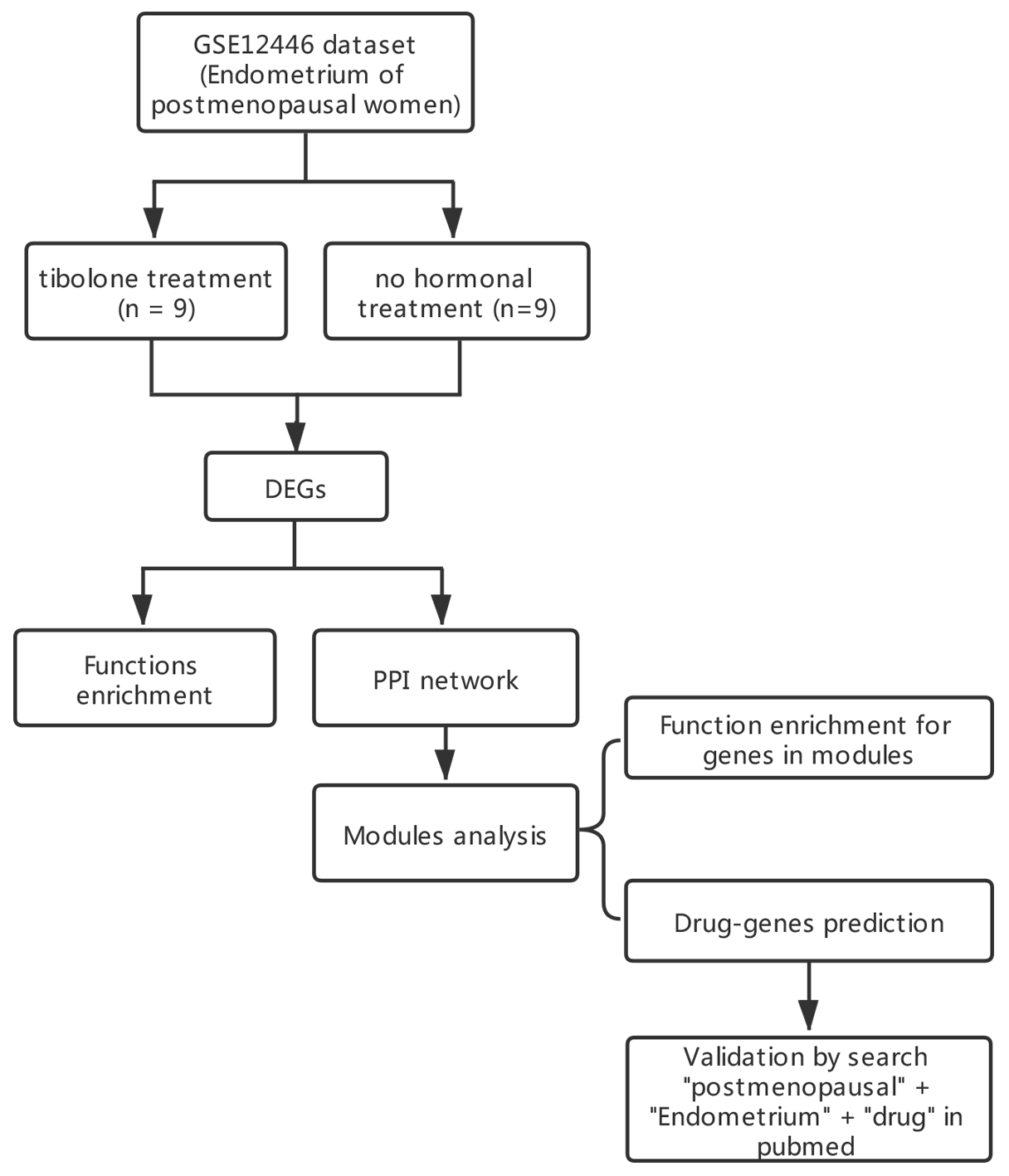

Supplement: Supplementary file 1 — Additional file 1: Figure S1. The workflow of this study. [file 12905_2020_1156_MOESM1_ESM.tif]
